# Supplementary material for: Phosphosite Scanning reveals a complex phosphorylation code underlying CDK-dependent activation of Hcm1
Source: Nat Commun. 2023 Jan 19;14:310. doi: 10.1038/s41467-023-36035-9 (PMC9852432; doi:10.1038/s41467-023-36035-9)
Supplement: Supplementary file 1 — Supplementary Information [file 41467_2023_36035_MOESM1_ESM.pdf]

Supplementary Information

Phosphosite Scanning reveals a complex phosphorylation code underlying CDK-dependent activation of Hcm1

Conti et al.

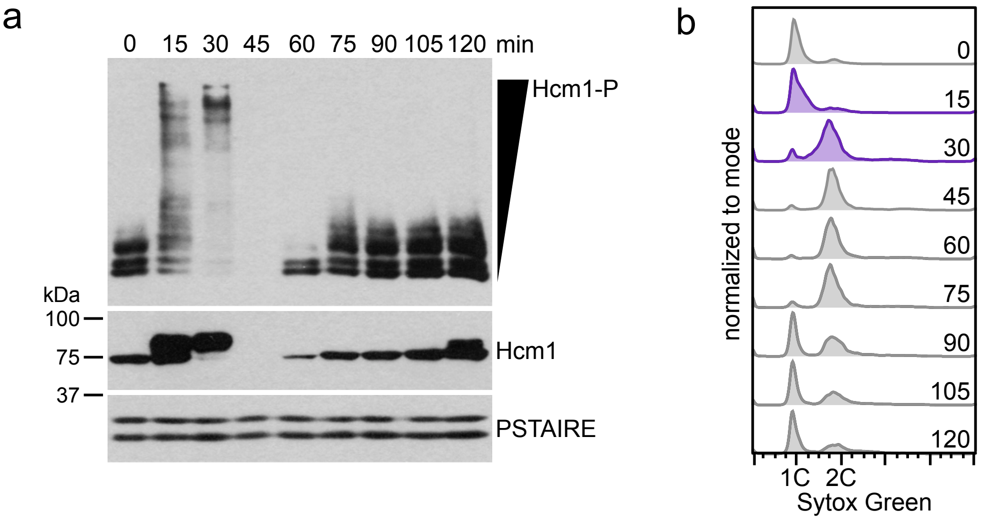

Supplementary Fig. 1. Phosphorylation of Hcm1 during the cell cycle. (a) Phos-tag Western blot showing Hcm1 phoshporylation during the cell cycle. Cells were arrested in G1 with alpha-factor and released into the cell cycle. Samples were taken after the indicated number of minutes. Alpha-factor was added back after 45 minutes to prevent cells from entering a second cycle. Hcm1 was detected with antibodies against the C-terminal 3V5 tag. PSTAIRE is shown as a loading control. Representative images from n = 3 biological replicates are shown. **(b)** Flow cytometry data showing DNA content that confirms cell cycle positions for the representative G1 arrest-release shown in part (a). Time points that include cells in S phase are shown in purple. 5,000 cells were analyzed from each time point.

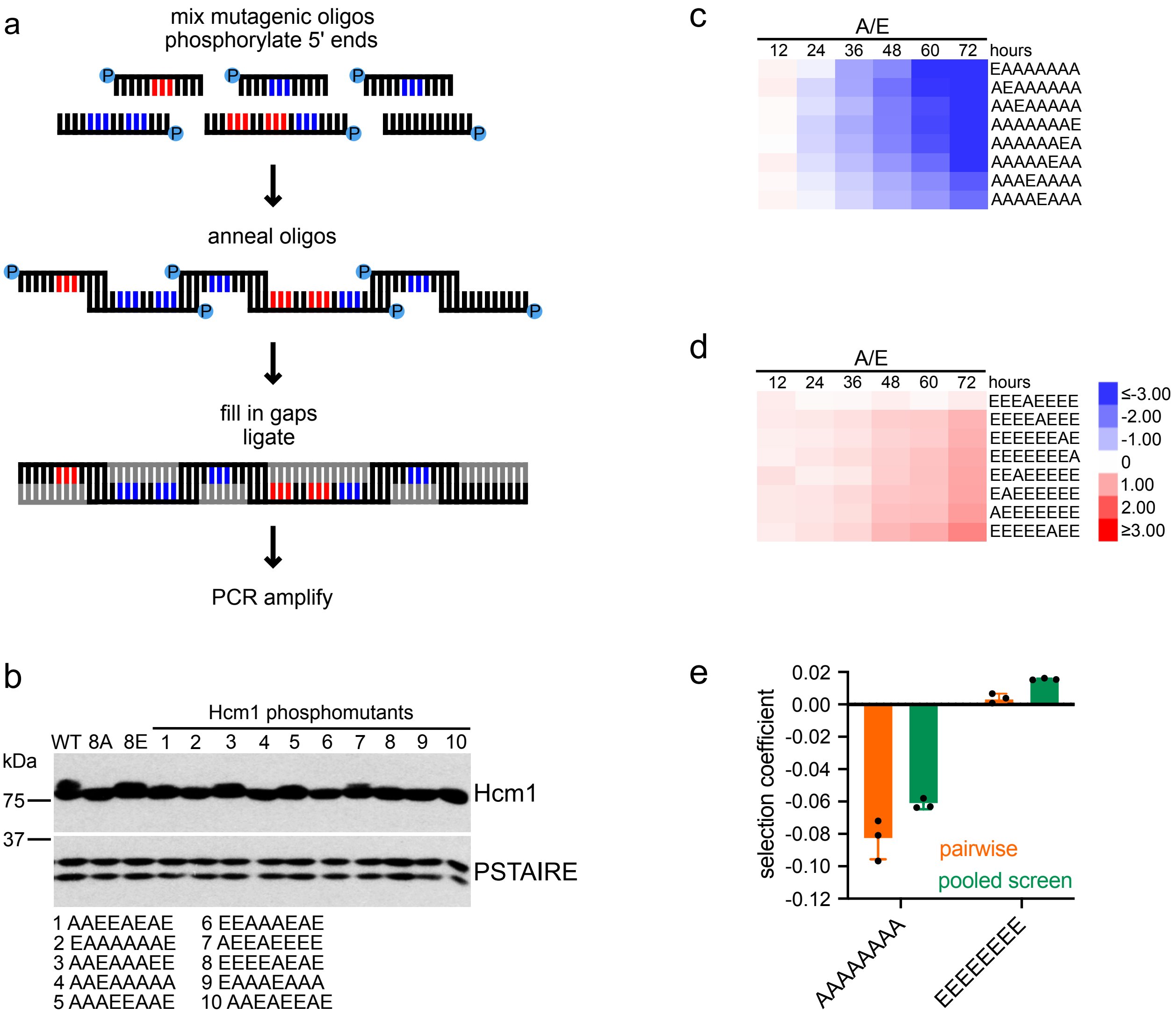

Supplementary Fig. 2. Supporting data for the A/E screen. (a) Schematic depicting the construction of phosphomutant plasmid libraries. Red and blue indicate point mutations to phosphosites. See Methods for more details. **(b)** Western blot of representative Hcm1 mutants and wild type (WT) proteins from the A/E screen. Hcm1 was detected using antibody recognizing a C-terminal 3V5 tag, PSTAIRE is shown as a loading control. Images from n = 1 biological replicate are shown. **(c-d)** Expanded view of 1E, 7A (c) and 7E, 1A (d) clusters from Fig. 2c. Each row represents a mutant with the indicated mutations, shown is the log2 fold change in normalized read counts with respect to time zero for each mutant, all mutants have been normalized to wild type. Blue indicates depletion, red indicates enrichment. Shown is an average of n = 3 biological replicates. Scale bar is the same for (c) and (d). **(e)** Average selection coefficents calculated from n = 3 replicates of pairwise assays in Fig. 1 (orange) and n = 3 replicates of the pooled A/E screen in Fig. 2 (green). Error bars represent standard deviations.

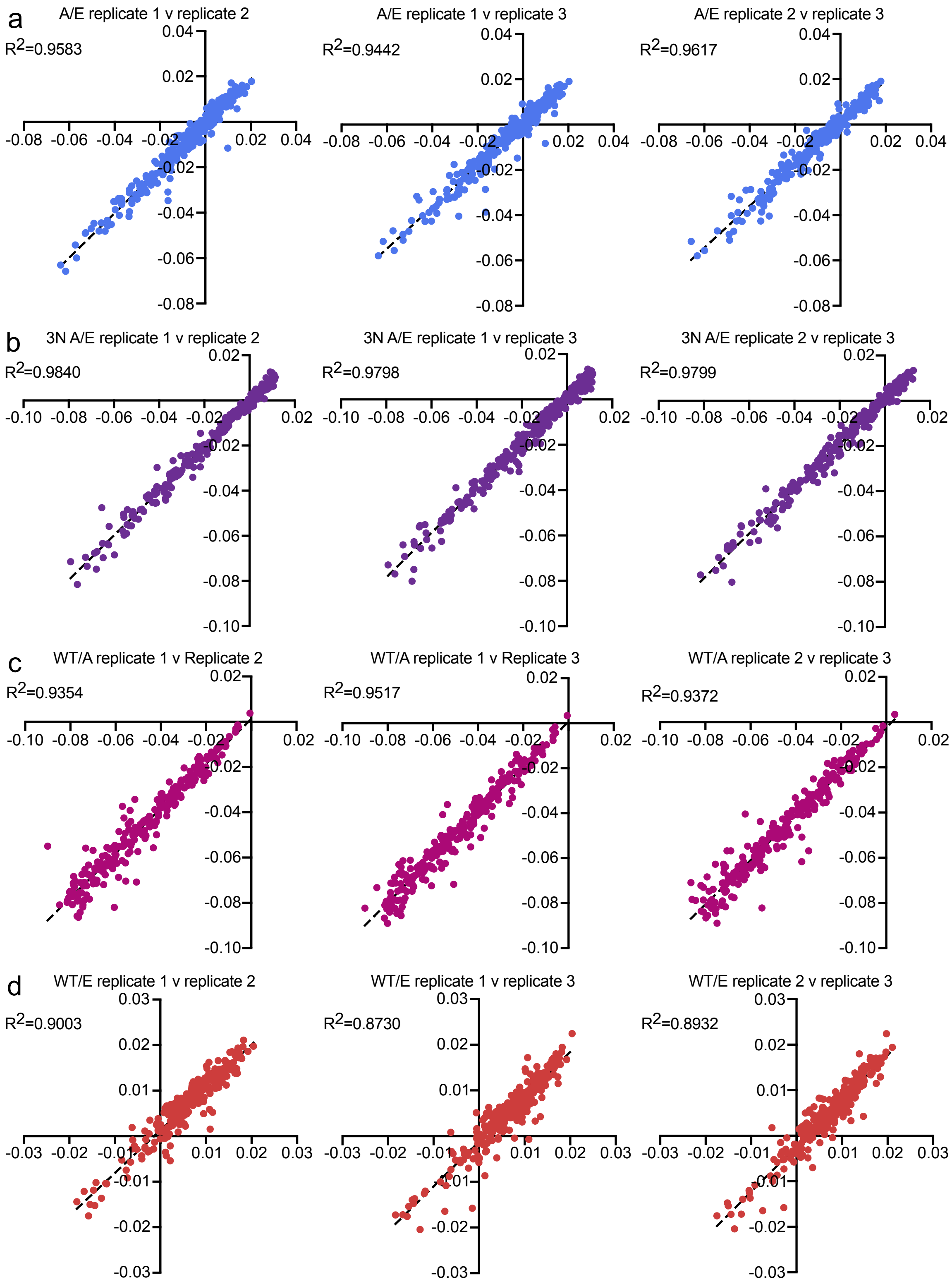

Supplementary Data Fig. 3. Replicate correlation plots for all screens. Scatter plots showing the correlation between independent replicates from the A/E **(a)**, 3N A/E **(b)**, WT/A **(c)**, WT/E **(d)** screens. R^2 values are shown with each plot.

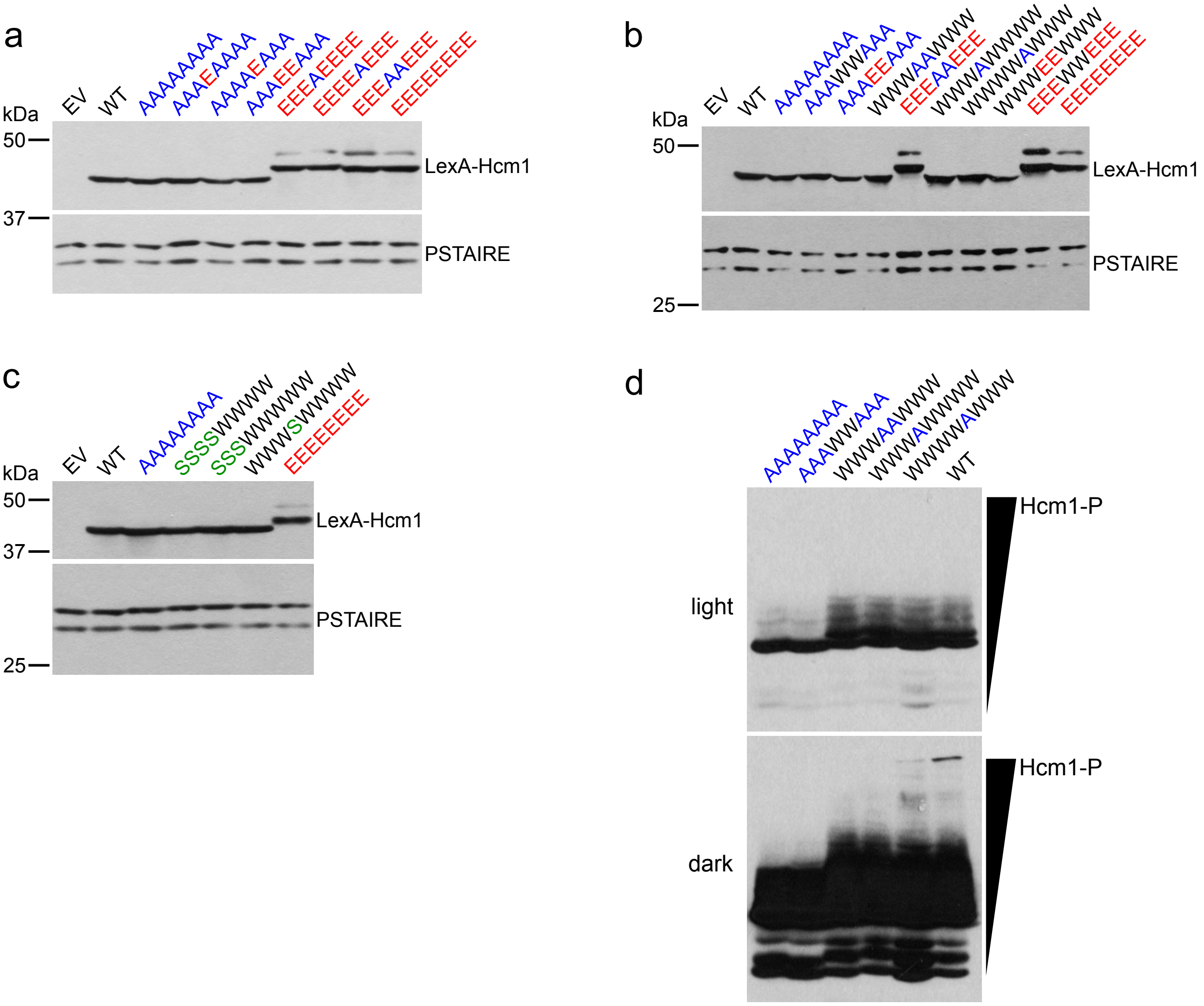

Supplementary Fig. 4. Expression and phosphorylation of LexA-Hcm1 fusion proteins. (a-c) Western blot showing expression of LexA-Hcm1 fusion constructs of the indicated genotypes to support Figs. 3a (a), 6c (b), and 7c (c). Wild type (WT) and empty vector (EV) controls are included. LexA-Hcm1 was detected using antibodies that detect a C-terminal 3V5 tag, PSTAIRE is shown as a loading control. Representative images from n=3 biological replicates each containing n=3 technical replicates are shown. **(d)** Phos-tag Western blot of in the indicated LexA-Hcm1 fusion proteins from (b). Light and dark exposures are shown. Representative images from n=3 biological replicates are shown.

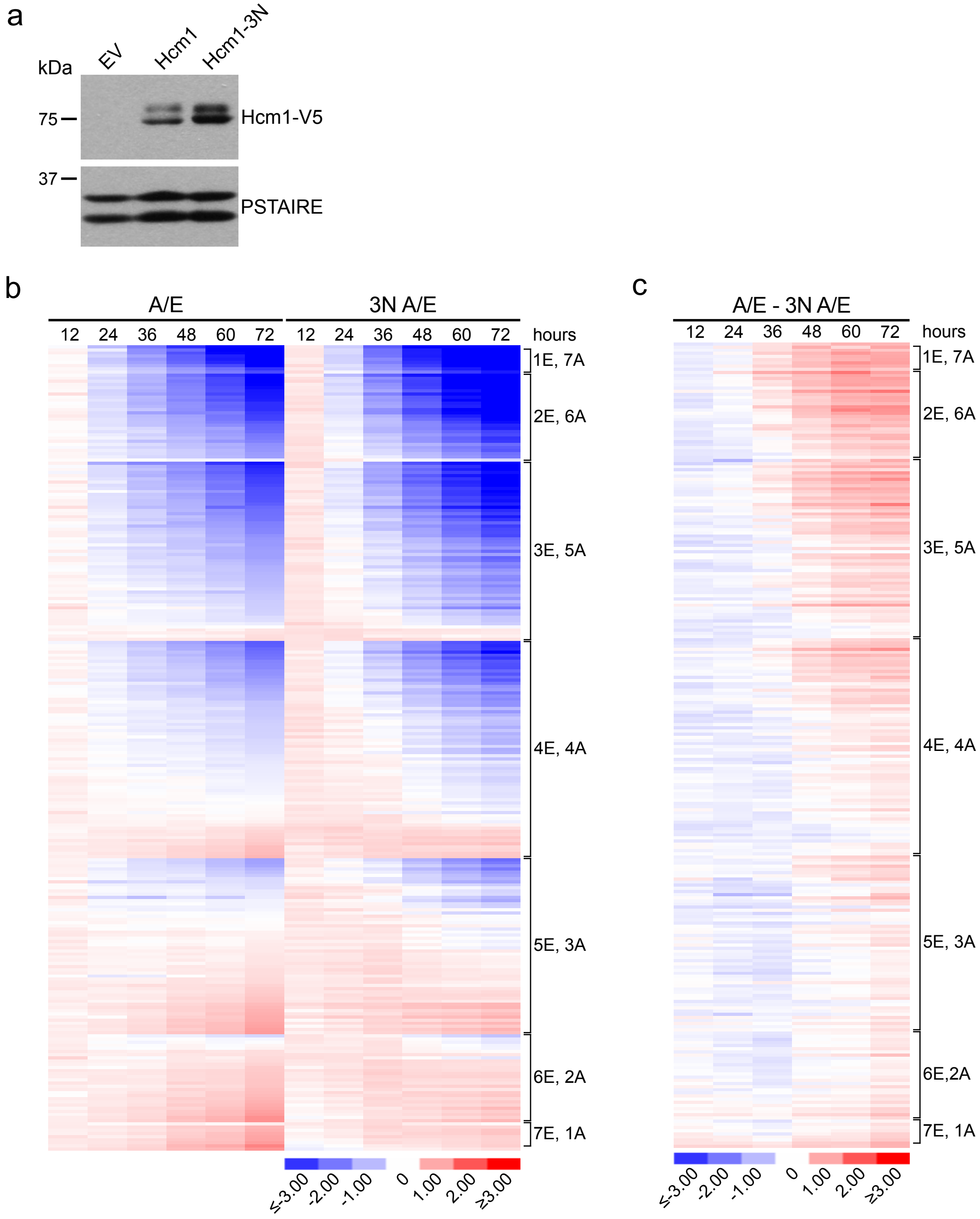

Supplementary Fig. 5. Supporting data for the 3N A/E screen. (a) Western blot showing expression of Hcm1 and Hcm1-3N from centromeric plasmids (pRS316). Hcm1 was detected using antibodies that recognize a C-terminal 3V5 tag, PSTAIRE is shown as a loading control. Representative images from n=2 biological replicates shown. **(b)** Side-by-side heat map representation of the results of the A/E and 3N A/E Phosphosite Scanning screens. Each row represents a mutant, shown is the log2 fold change in normalized read counts with respect to time zero for each mutant. In the A/E screen all mutants were normalized to WT. In the 3N A/E screen all mutants are normalized to *hcm1-3N*. Blue indicates depletion, red indicates enrichment. Shown is an average of n = 3 biological replicates. **(c)** Heat map representation of the difference between heat maps in the A/E and 3N A/E screens (from part b). Blue indicates normalized read counts were higher in 3N A/E screen, red indicates normalized read counts were higher in the A/E screen.

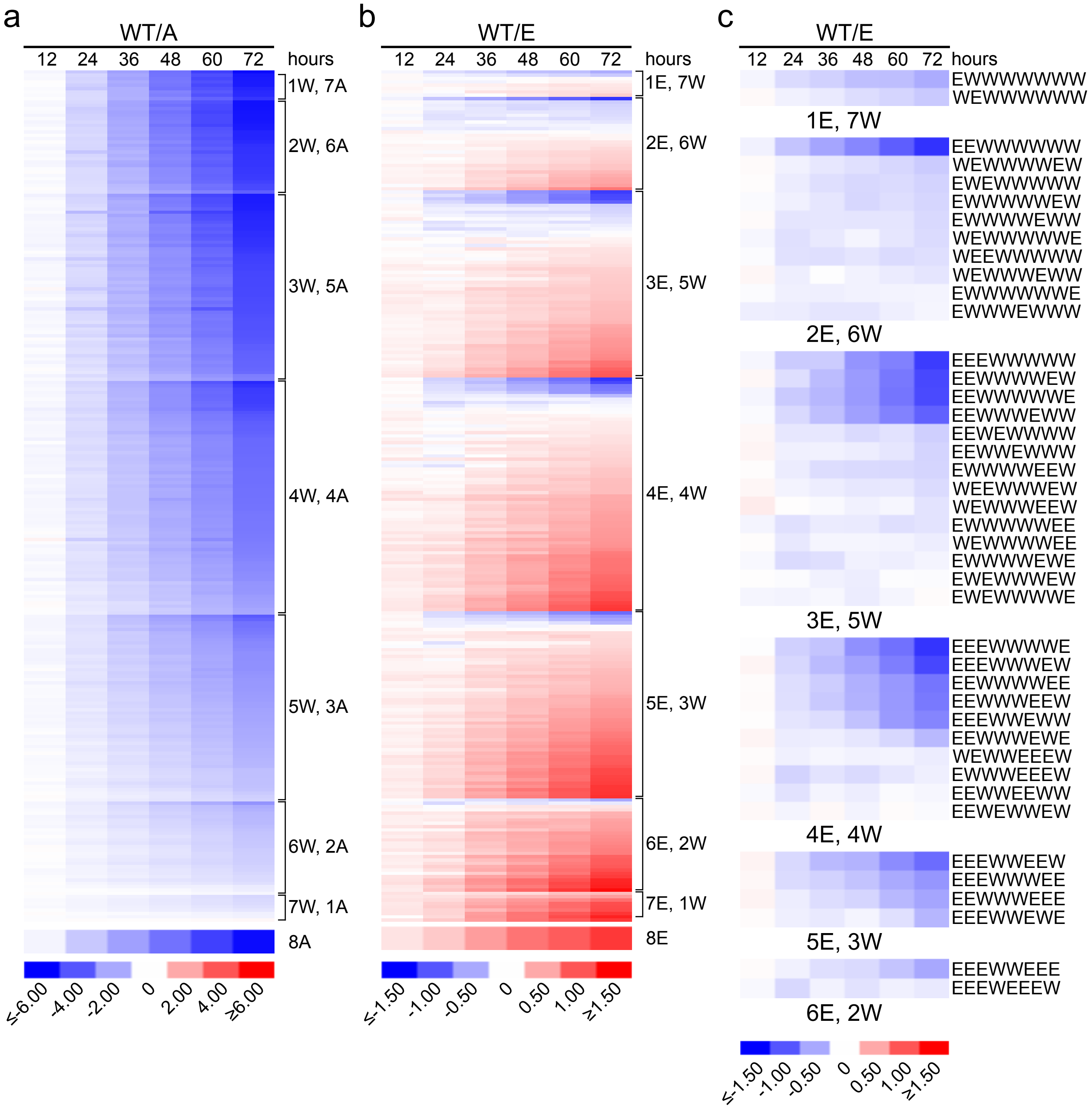

Supplementary Fig. 6. Supporting data for the WT/A and WT/E screens. (a-b) Heat map representation of the results of the WT/A (a) and WT/E (b) Phosphosite Scanning screens. Each row represents a mutant, shown is the log2 fold change in normalized read counts with respect to time zero for each mutant, all mutants have been normalized to wild type. Blue indicates depletion, red indicates enrichment. Shown are averages of n = 3 biological replicates. Note difference in scale bars between (a) and (b). **(c)** Expanded view of partial clusters from (b) that include mutants selected against over time. Blue indicates depletion red indicates enrichment. Note that all mutants in this category have phosphomimetic mutations in the first and/or second position.

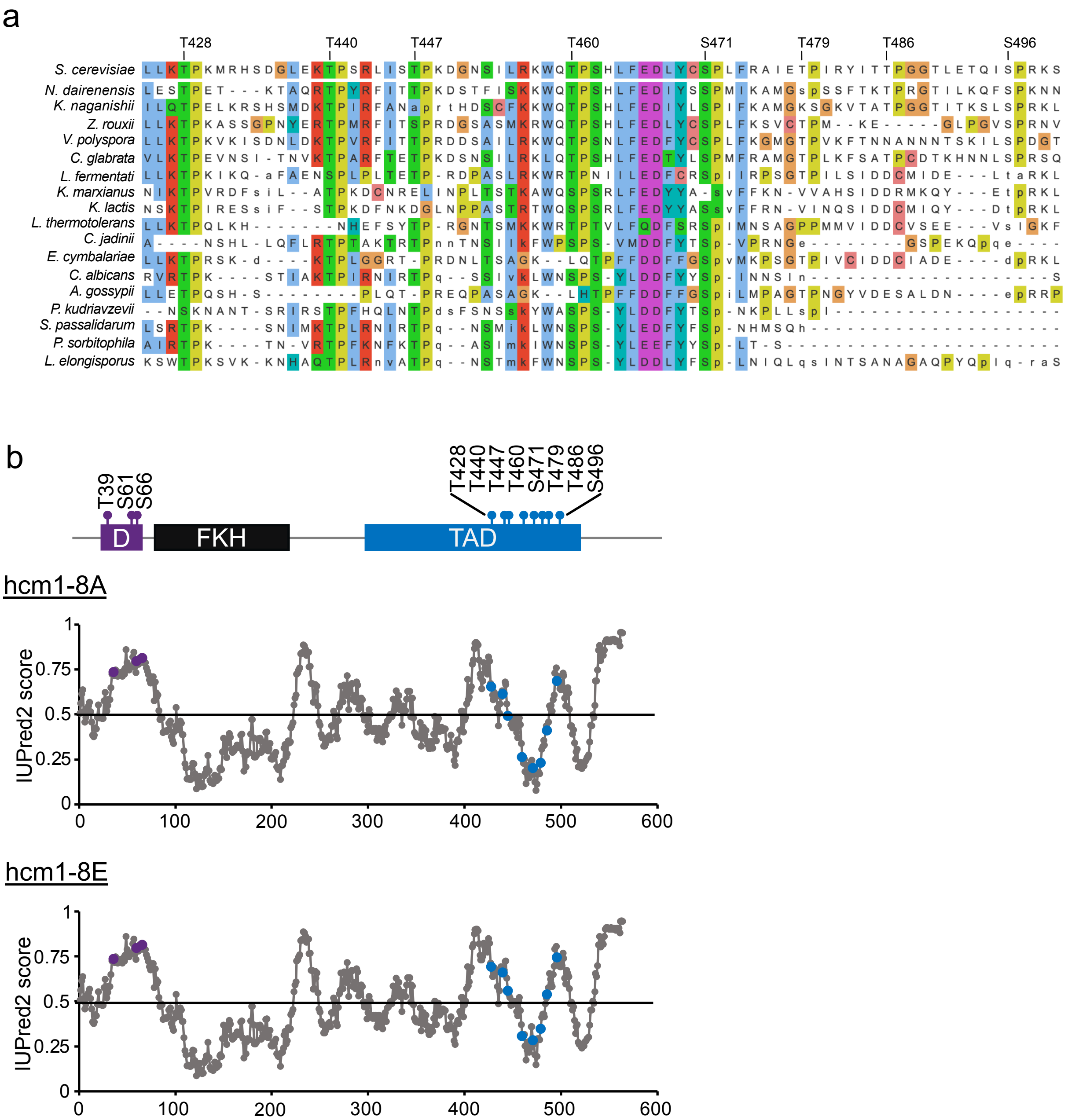

Supplementary Fig. 7. Conservation and structure of Hcm1. (a) Multiple sequence alignment of the Hcm1 TAD from budding yeasts. Hcm1 sequences from the indicated species was visualized using ProViz (http://slim.icr.ac.uk/proviz/). The region shown corresponds to amino acids 425 through 500 in *S. cerevisiae* Hcm1. CDK phosphosites investigated in this study are indicated. **(b)** Predicted disorder was calcuated using IUPred2 for hcm1-8A and hcm1-8E mutant proteins. The WT Hcm1 IUPred2 prediction is shown in Fig 1a.

Supplementary Table 1. Strain table.

Strain name	Genotype	Figure
YMC50	*MATa his3Δ1 ura3Δ0 leu2Δ0 lys2Δ0 HIS3MX6-GAL1p-HCM1-3HA-KanMX ChrVIΔ181901-182001::Hyg-TEFp-GFP + pRS316-HCM1p-HCM1-3V5*	1b,c, 4a, S2e
YMC53	*MATa his3Δ1 ura3Δ0 leu2Δ0 lys2Δ0 HIS3MX6-GAL1p-HCM1-3HA-KanMX ChrVIΔ181901-182001::Hyg-TEFp-GFP(Y66F) + pRS316-HCM1p-HCM1-3V5*	1b-e, S2e
YMC51	*MATa his3Δ1 ura3Δ0 leu2Δ0 lys2Δ0 HIS3MX6-GAL1p-HCM1-3HA-KanMX ChrVIΔ181901-182001::Hyg-TEFp-GFP + pRS316-HCM1p-hcm1-8A-3V5*	1d, S2e
YMC52	*MATa his3Δ1 ura3Δ0 leu2Δ0 lys2Δ0 HIS3MX6-GAL1p-HCM1-3HA-KanMX ChrVIΔ181901-182001::Hyg-TEFp-GFP + pRS316-HCM1p-hcm1-8E-3V5*	1e, S2e
YMC9	*MATa his3Δ1 ura3Δ0 leu2Δ0 lys2Δ0 HIS3MX6-GAL1p-HCM1-3HA-KanMX*	2c-g, 4e-g, 5b, c, e, f, 6a-b, S2c-e, S3, S5b-c, S6
YMC390	*MATa ade2 his3 leu2 trp1 LYS2::lexAop-HIS3 URA3::lexAop-lacZ + pBTM116*	3a, 6c, 7c, S4a-c
YMC401	*MATa ade2 his3 leu2 trp1 LYS2::lexAop-HIS3 URA3::lexAop-lacZ + pBTM116-hcm1(306-511)-3V5*	3a, 6c, 7c, S4
YMC402	*MATa ade2 his3 leu2 trp1 LYS2::lexAop-HIS3 URA3::lexAop-lacZ + pBTM116-hcm1(306-511)-8A-3V5*	3a, 6c, 7c, S4
YMC403	*MATa ade2 his3 leu2 trp1 LYS2::lexAop-HIS3 URA3::lexAop-lacZ + pBTM116-hcm1(306-511)-8E-3V5*	3a, 6c, 7c, S4a-c
YMC395	*MATa ade2 his3 leu2 trp1 LYS2::lexAop-HIS3 URA3::lexAop-lacZ + pBTM116-hcm1(306-511)-AAAEAAAA-3V5*	3a, S4a
YMC396	*MATa ade2 his3 leu2 trp1 LYS2::lexAop-HIS3 URA3::lexAop-lacZ + pBTM116-hcm1(306-511)-AAAAEAAA-3V5*	3a, S4a
YMC397	*MATa ade2 his3 leu2 trp1 LYS2::lexAop-HIS3 URA3::lexAop-lacZ + pBTM116-hcm1(306-511)-AAAEEAAA-3V5*	3a, 6c, S4a-b
YMC398	*MATa ade2 his3 leu2 trp1 LYS2::lexAop-HIS3 URA3::lexAop-lacZ + pBTM116-hcm1(306-511)-EEEAEEEE-3V5*	3a, S4a
YMC399	*MATa ade2 his3 leu2 trp1 LYS2::lexAop-HIS3 URA3::lexAop-lacZ + pBTM116-hcm1(306-511)-EEEEAEEE-3V5*	3a, S4a
YMC400	*MATa ade2 his3 leu2 trp1 LYS2::lexAop-HIS3 URA3::lexAop-lacZ + pBTM116-hcm1(306-511)-EEEAAEEE-3V5*	3a, 6c, S4a-b
YBL192	*MATa his3Δ1 ura3Δ0 leu2Δ0 met15Δ0 HCM1-3V5-KanMX*	3b-d, 6d, 7b, S1
YMC360	*MATa his3Δ1 ura3Δ0 leu2Δ0 met15Δ0 hcm1-8A-3V5-KanMX*	3b-d, 6d, 7b
YMC326	*MATa his3Δ1 ura3Δ0 leu2Δ0 met15Δ0 hcm1-AAAEEAAA-3V5-KanMX*	3b-d
YMC359	*MATa his3Δ1 ura3Δ0 leu2Δ0 met15Δ0 hcm1-EEEAAEEE-3V5-KanMX*	3b-d
YMC356	*MATa his3Δ1 ura3Δ0 leu2Δ0 met15Δ0 hcm1-8E-3V5-KanMX*	3b-d
YMC324	*MATa his3Δ1 ura3Δ0 leu2Δ0 met15Δ0 hcm1-AAAEAAAA-3V5-KanMX*	3b-d
YMC325	*MATa his3Δ1 ura3Δ0 leu2Δ0 met15Δ0 hcm1-AAAAEAAA-3V5-KanMX*	3b-d
YMC357	*MATa his3Δ1 ura3Δ0 leu2Δ0 met15Δ0 hcm1-EEEAEEEE-3V5-KanMX*	3b-d
YMC358	*MATa his3Δ1 ura3Δ0 leu2Δ0 met15Δ0 hcm1-EEEEAEEE-3V5-KanMX*	3b-d
YMC443	*MATa his3Δ1 ura3Δ0 leu2Δ0 lysΔ0 HIS3MX6-GAL1p-HCM1-3HA-KanMX ChrVIΔ181901-182001::HYG-TEFp-GFP + pRS316-HCM1p-hcm1-3N-3V5*	4b-c

YMC446	*MATa his3Δ1 ura3Δ0 leu2Δ0 lys2Δ0 HIS3MX6-GAL1p-HCM1-3HA-KanMX ChrVIΔ181901-182001::Hyg-TEFp-GFP(Y66F) + pRS316-HCM1p-hcm1-3N-3V5*	4a
YMC448	*MATa his3Δ1 ura3Δ0 leu2Δ0 lys2Δ0 HIS3MX6-GAL1p-HCM1-3HA-KanMX ChrVIΔ181901-182001::Hyg-TEFp-GFP(Y66F) + pRS316-HCM1p-hcm1-3N8E-3V5*	4c
YMC447	*MATa his3Δ1 ura3Δ0 leu2Δ0 lys2Δ0 HIS3MX6-GAL1p-HCM1-3HA-KanMX ChrVIΔ181901-182001::Hyg-TEFp-GFP(Y66F) + pRS316-HCM1p-hcm1-3N8A-3V5*	4b
YMN1	*MATa ade2 his3 leu2 trp1 LYS2::lexAop-HIS3 URA3::lexAop-lacZ + pBTM116-hcm1(306-511)-WWWEEWWW-3V5*	6c, S4b
YMN2	*MATa ade2 his3 leu2 trp1 LYS2::lexAop-HIS3 URA3::lexAop-lacZ + pBTM116-hcm1(306-511)-EEEWWEEE-3V5*	6c, S4b
YMC435	*MATa ade2 his3 leu2 trp1 LYS2::lexAop-HIS3 URA3::lexAop-lacZ + pBTM116-hcm1(306-511)-WWWAAWWW-3V5*	6c, S4b,d
YMC434	*MATa ade2 his3 leu2 trp1 LYS2::lexAop-HIS3 URA3::lexAop-lacZ + pBTM116-hcm1(306-511)-AAAWWAAA-3V5*	6c, S4b,d
YMC438	*MATa ade2 his3 leu2 trp1 LYS2::lexAop-HIS3 URA3::lexAop-lacZ + pBTM116-hcm1(306-511)-WWWAWWWW-3V5*	6c, S4b,d
YMC439	*MATa ade2 his3 leu2 trp1 LYS2::lexAop-HIS3 URA3::lexAop-lacZ + pBTM116-hcm1(306-511)-WWWWAWWW-3V5*	6c, S4b,d
YBL193	*MATa his3Δ1 ura3Δ0 leu2Δ0 met15Δ0 hcm1-15A-3V5-KanmX*	6d,e, 7b
YMC426	*MATa his3Δ1 ura3Δ0 leu2Δ0 met15Δ0 hcm1-AAAWAAAA-3V5-KanMX*	6d
YMC427	*MATa his3Δ1 ura3Δ0 leu2Δ0 met15Δ0 hcm1-AAAAWAAA-3V5-KanMX*	6d
YMC423	*MATa his3Δ1 ura3Δ0 leu2Δ0 met15Δ0 hcm1-AAAWWAAA-3V5-KanMX*	6d, 7b
YMC425	*MATa his3Δ1 ura3Δ0 leu2Δ0 met15Δ0 hcm1-WWWAAWWW-3V5-KanMX*	6d
YMC428	*MATa his3Δ1 ura3Δ0 leu2Δ0 met15Δ0 hcm1-WWWAWWWW-3V5-KanMX*	6d
YMC429	*MATa his3Δ1 ura3Δ0 leu2Δ0 met15Δ0 hcm1-WWWWAWWW-3V5-KanMX*	6d
YMC581	*MATa his3Δ1 ura3Δ0 leu2Δ0 met15Δ0 hcm1-7N+AAAWWAAA-3V5-KanMX*	6e
YMC585	*MATa his3Δ1 ura3Δ0 leu2Δ0 met15Δ0 hcm1-7N+AAAWWWWW-3V5-KanMX*	6e
YHA163	*MATa his3Δ1 ura3Δ0 leu2Δ0 met15Δ0 hcm1-7N-3V5-KanMX*	6e
YMC430	*MATa his3Δ1 ura3Δ0 leu2Δ0 met15Δ0 hcm1-SSSSWWWW-3V5-KanMX*	7b
YMC431	*MATa his3Δ1 ura3Δ0 leu2Δ0 met15Δ0 hcm1-SSSWWWWW-3V5-KanMX*	7b
YMC432	*MATa his3Δ1 ura3Δ0 leu2Δ0 met15Δ0 hcm1-WWWSWWWW-3V5-KanMX*	7b
YMC440	*MATa ade2 his3 leu2 trp1 LYS2::lexAop-HIS3 URA3::lexAop-lacZ + pBTM116-hcm1(306-511)-SSSSWWWW-3V5*	7c, S4c
YMC441	*MATa ade2 his3 leu2 trp1 LYS2::lexAop-HIS3 URA3::lexAop-lacZ + pBTM116-hcm1(306-511)-SSSWWWWW-3V5*	7c, S4c
YMC442	*MATa ade2 his3 leu2 trp1 LYS2::lexAop-HIS3 URA3::lexAop-lacZ + pBTM116-hcm1(306-511)-WWWSWWWW-3V5*	7c, S4c
YMC343	*MATa his3Δ1 ura3Δ0 leu2Δ0 met15Δ0 + pRS316-HCM1p-HCM1-3V5*	S2b
YMC344	*MATa his3Δ1 ura3Δ0 leu2Δ0 met15Δ0 + pRS316-HCM1p-hcm1-8A-3V5*	S2b
YMC345	*MATa his3Δ1 ura3Δ0 leu2Δ0 met15Δ0 + pRS316-HCM1p-hcm1-8E-3V5*	S2b
YMC346	*MATa his3Δ1 ura3Δ0 leu2Δ0 met15Δ0 + pRS316-HCM1p-hcm1-AAEEAEAE-3V5*	S2b

YMC347	*MATa his3Δ0 ura3Δ0 leu2Δ0 met15Δ0 + pRS316-HCM1p-hcm1-EAAAAAAE-3V5*	S2b
YMC348	*MATa his3Δ1 ura3Δ0 leu2Δ0 met15Δ0 + pRS316-HCM1p-hcm1-AAEAAAEE-3V5*	S2b
YMC349	*MATa his3Δ1 ura3Δ0 leu2Δ0 met15Δ0 + pRS316-HCM1p-hcm1-AAEAAAAA-3V5*	S2b
YMC350	*MATa his3Δ1 ura3Δ0 leu2Δ0 met15Δ0 + pRS316-HCM1p-hcm1-AAAEEAAE-3V5*	S2b
YMC351	*MATa his3Δ1 ura3Δ0 leu2Δ0 met15Δ0 + pRS316-HCM1p-hcm1-EEAAAEAE-3V5*	S2b
YMC352	*MATa his3Δ1 ura3Δ0 leu2Δ0 met15Δ0 + pRS316-HCM1p-hcm1-AEEAEEEE-3V5*	S2b
YMC353	*MATa his3Δ0 ura3Δ0 leu2Δ0 met15Δ0 + pRS316-HCM1p-hcm1-EEEEAEAE-3V5*	S2b
YMC354	*MATa his3Δ1 ura3Δ0 leu2Δ0 met15Δ0 + pRS316-HCM1p-hcm1-EAAAEAAA-3V5*	S2b
YMC355	*MATa his3Δ1 ura3Δ0 leu2Δ0 met15Δ0 + pRS316-HCM1p-hcm1-AAEAEEAE-3V5*	S2b
YMC454	*MATa his3Δ1 ura3Δ0 leu2Δ0 met15Δ0 hcm1Δ::KanMX + pRS316*	S5a
YMC455	*MATa his3Δ1 ura3Δ0 leu2Δ0 met15Δ0 hcm1Δ::KanMX + pRS316-HCM1-3V5*	S5a
YMC458	*MATa his3Δ1 ura3Δ0 leu2Δ0 met15Δ0 hcm1Δ::KanMX + pRS316-hcm1-3N-3V5*	S5a

Supplementary Table 2. Plasmid table.

Plasmid name	Description	Figure
pRS316-HCM1-3V5	*HCM1p-HCM1-3V5*, CEN, URA3	1b-e, 4a, S2b,e, S5a,
pRS316-hcm1-8A-3V5	*HCM1p-hcm1-8A-3V5*, CEN, URA3	1d, S2b,e
pRS316-hcm1-8E-3V5	*HCM1p-hcm1-8E-3V5*, CEN, URA3	1e, S2b,e
pRS316-hcm1-3N-3V5	*HCM1p-hcm1-3N-3V5,* CEN, URA3	4a-c, S5a
pRS316-hcm1-3N8A-3V5	*HCM1p-hcm1-3N8A-3V5*, CEN, URA3	4b
pRS316-hcm1-3N8E-3V5	*HCM1p-hcm1-3N8E-3V5*, CEN, URA3	4c
pBTM116	*ADH1p-LEXA*, 2micron, TRP	3a, 6c, 7c, S4a-c
pBTM116-hcm1(306-511)-3V5	*ADH1p-LEXA-hcm1(306-511)-3V5*, 2micron, TRP	3a, 6c, 7c, S4a-d
pBTM116-hcm1(306-511)-8A-3V5	*ADH1p-LEXA-hcm1(306-511)-8A-3V5*, 2micron, TRP	3a, 6c, 7c, S4a-d
pBTM116-hcm1(306-511)-AAAEAAAA-3V5	*ADH1p-LEXA-hcm1(306-511)-AAAEAAAA-3V5*, 2micron, TRP	3a, S4a
pBTM116-hcm1(306-511)-AAAAEAAA-3V5	*ADH1p-LEXA-hcm1(306-511)-AAAAEAAA-3V5*, 2micron, TRP	3a, S4a
pBTM116-hcm1(306-511)-AAAEEAAA-3V5	*ADH1p-LEXA-hcm1(306-511)-AAAEEAAA-3V5*, 2micron, TRP	3a, 6c, S4a-b
pBTM116-hcm1(306-511)-EEEAEEEE-3V5	*ADH1p-LEXA-hcm1(306-511)-EEEAEEEE-3V5*, 2micron, TRP	3a, S4a
pBTM116-hcm1(306-511)-EEEEAEEE-3V5	*ADH1p-LEXA-hcm1(306-511)-EEEEAEEE-3V5*, 2micron, TRP	3a, S4a
pBTM116-hcm1(306-511)-EEEAAEEE-3V5	*ADH1p-LEXA-hcm1(306-511)-EEEAAEEE-3V5*, 2micron, TRP	3a, 6c, S4a-b
pBTM116-hcm1(306-511)-8E-3V5	*ADH1p-LEXA-hcm1(306-511)-8E-3V5*, 2micron, TRP	3a, 6c, 7c, S4a-c
pBTM116-hcm1(306-511)-AAAWWAAA-3V5	*ADH1p-LEXA-hcm1(306-511)-AAAWWAAA-3V5*, 2micron, TRP	6c, S4b,d
pBTM116-hcm1(306-511)-WWWAAWWW-3V5	*ADH1p-LEXA-hcm1(306-511)-WWWAAWWW-3V5*, 2micron, TRP	6c, S4b,d
pBTM116-hcm1(306-511)-WWWAWWWW-3V5	ADH1p-LEXA-hcm1(306-511)-WWWAWWWW-3V5, 2micron, TRP	6c, S4b,d
pBTM116-hcm1(306-511)-WWWWAWWW-3V5	*ADH1p-LEXA-hcm1(306-511)-WWWWAWWW-3V5*, 2micron, TRP	6c, S4b,d
pBTM116-hcm1(306-511)-WWWEEWWW-3V5	*ADH1p-LEXA-hcm1(306-511)-WWWEEWWW-3V5*, 2micron, TRP	6c, S4b
pBTM116-hcm1(306-511)-EEEWWEEE-3V5	*ADH1p-LEXA-hcm1(306-511)-EEEWWEEE-3V5*, 2micron, TRP	6c, S4b
pBTM116-hcm1(306-511)-SSSSWWWW-3V5	*ADH1p-LEXA-hcm1(306-511)-SSSSWWWW-3V5*, 2micron, TRP	7c, S4c
pBTM116-hcm1(306-511)-SSSWWWWW-3V5	*ADH1p-LEXA-hcm1(306-511)-SSSWWWWW-3V5*, 2micron, TRP	7c, S4c
pBTM116-hcm1(306-511)-WWWSWWWW-3V5	*ADH1p-LEXA-hcm1(306-511)-WWWSWWWW-3V5*, 2micron, TRP	7c, S4c
pRS316-hcm1-AAEEAEAE-3V5	*HCM1p-hcm1-AAEEAEAE-3V5*, CEN, URA3	S2b
pRS316-hcm1-EAAAAAAE-3V5	*HCM1p-hcm1-EAAAAAAE-3V5*, CEN, URA3	S2b

pRS316-hcm1-AAEAAAEE-3V5	*HCM1p-hcm1-AAEAAAEE-3V5*, CEN, URA3	S2b
pRS316-hcm1-AAEAAAAA-3V5	*HCM1p-hcm1-AAEAAAAA-3V5*, CEN, URA3	S2b
pRS316-hcm1-AAAEEAAE-3V5	*HCM1p-hcm1-AAAEEAAE-3V5*, CEN, URA3	S2b
pRS316-hcm1-EEAAAEAE-3V5	*HCM1p-hcm1-EEAAAEAE-3V5*, CEN, URA3	S2b
pRS316-hcm1-AEEAEEEE-3V5	*HCM1p-hcm1-AEEAEEEE-3V5*, CEN, URA3	S2b
pRS316-hcm1-EEEEAEAE-3V5	*HCM1p-hcm1-EEEEAEAE-3V5*, CEN, URA3	S2b
pRS316-hcm1-EAAAEAAA-3V5	*HCM1p-hcm1-EAAAEAAA-3V5*, CEN, URA3	S2b
pRS316-hcm1-AAEAEEAE-3V5	*HCM1p-hcm1-AAEAEEAE-3V5*, CEN, URA3	S2b
pRS316-hcm1-A/E	*HCM1p-hcm1-A/E-3V5,* CEN, URA3	2c-g, 4g, 6a-b, S2c-e, S3a, S5b-c
pRS316-hcm1-3NA/E	*HCM1p-hcm1-3NA/E-3V5,* CEN, URA3	4e-g, S3b, S5b-c
pRS316-hcm1-WT/A	*HCM1p-hcm1-WT/A-3V5,* CEN, URA3	5b-c, 6b, S3c, S6a
pRS316-hcm1-WT/E	*HCM1p-hcm1-WT/E-3V5,* CEN, URA3	5e-f, 6a-b, S3d, S6b-c

Supplementary Table 3. Oligonucleotides for mutagenesis.

Name	Sites mutated	Genotype	orientation	Sequence
A1	T428	A	FWD	ccttcctctcatggttcggacttacttaaa**GCTCCT**aagatgaggcattccgatggcttagagaaa
A2	T428	E	FWD	ccttcctctcatggttcggacttacttaaa**GAGGAA**aagatgaggcattccgatggcttagagaaa
A3	T428	W	FWD	ccttcctctcatggttcggacttacttaaa**ACACCC**aagatgaggcattccgatggcttagagaaa
B1	T440, T447	AA	REV	ctgccatttcctcaaaatcgagttaccgtcctt**TGGCGC**gcttatcaaccgcga**CGGAGC**tttctctaagccatcggaatgc
B2	T440, T447	AE	REV	ctgccatttcctcaaaatcgagttaccgtcctt**CTCTTC**gcttatcaaccgcga**CGGAGC**tttctctaagccatcggaatgc
B3	T440, T447	EA	REV	ctgccatttcctcaaaatcgagttaccgtcctt**TGGCGC**gcttatcaaccgcga**TTCCTC**tttctctaagccatcggaatgc
B4	T440, T447	EE	REV	ctgccatttcctcaaaatcgagttaccgtcctt**CTCTTC**gcttatcaaccgcga**TTCCTC**tttctctaagccatcggaatgc
B5	T440, T447	AW	REV	ctgccatttcctcaaaatcgagttaccgtcctt**AGGTGT**gcttatcaaccgcga**CGGAGC**tttctctaagccatcggaatgc
B6	T440, T447	WA	REV	ctgccatttcctcaaaatcgagttaccgtcctt**TGGCGC**gcttatcaaccgcga**TGGGGT**tttctctaagccatcggaatgc
B7	T440, T447	WW	REV	ctgccatttcctcaaaatcgagttaccgtcctt**AGGTGT**gcttatcaaccgcga**TGGGGT**tttctctaagccatcggaatgc
B8	T440, T447	WE	REV	ctgccatttcctcaaaatcgagttaccgtcctt**CTCTTC**gcttatcaaccgcga**TGGGGT**tttctctaagccatcggaatgc
B9	T440, T447	EW	REV	ctgccatttcctcaaaatcgagttaccgtcctt**AGGTGT**gcttatcaaccgcga**TTCCTC**tttctctaagccatcggaatgc
C1	T460	A	FWD	actcgattttgaggaaatggcag**GCACCA**tcacacctttttgaagatttgtactgt
C2	T460	E	FWD	actcgattttgaggaaatggcag**GAGGAG**tcacacctttttgaagatttgtactgt
C3	T460	W	FWD	actcgattttgaggaaatggcag**ACTCCT**tcacacctttttgaagatttgtactgt
D1	S471, T479, T486	AAA	REV	aatttgggtttccaaagtgccccc**GGGGGC**cgtgatatacctgat**AGGAGC**ctctatagctctaaatag**GGGTGC**acagtacaaatcttcaaaaaggtgtga
D2	S471, T479, T486	AAE	REV	aatttgggtttccaaagtgccccc**TTCCTC**cgtgatatacctgat**AGGAGC**ctctatagctctaaatag**GGGTGC**acagtacaaatcttcaaaaaggtgtga
D3	S471, T479, T486	AEA	REV	aatttgggtttccaaagtgccccc**GGGGGC**cgtgatatacctgat**CTCTTC**ctctatagctctaaatag**GGGTGC**acagtacaaatcttcaaaaaggtgtga
D4	S471, T479, T486	AEE	REV	aatttgggtttccaaagtgccccc**TTCCTC**cgtgatatacctgat**CTCTTC**ctctatagctctaaatag**GGGTGC**acagtacaaatcttcaaaaaggtgtga
D5	S471, T479, T486	EAA	REV	aatttgggtttccaaagtgccccc**GGGGGC**cgtgatatacctgat**AGGAGC**ctctatagctctaaatag**TTCCTC**acagtacaaatcttcaaaaaggtgtga
D6	S471, T479, T486	EAE	REV	aatttgggtttccaaagtgccccc**TTCCTC**cgtgatatacctgat**AGGAGC**ctctatagctctaaatag**TTCCTC**acagtacaaatcttcaaaaaggtgtga
D7	S471, T479, T486	EEA	REV	aatttgggtttccaaagtgccccc**GGGGGC**cgtgatatacctgat**CTCTTC**ctctatagctctaaatag**TTCCTC**acagtacaaatcttcaaaaaggtgtga

D8	S471, T479, T486	EEE	REV	aatttgggtttccaaagtgcccccTTCCTCcgtgatatacctgatCTCTTCctctatagctctaaatagTTCCTCacagtacaaatcttcaaaaaggtgtga
D9	S471, T479, T486	AAW	REV	aatttgggtttccaaagtgcccccCGGCGTcgtgatatacctgatAGGAGCctctatagctctaaatagGGGTGCacagtacaaatcttcaaaaaggtgtga
D10	S471, T479, T486	AWA	REV	aatttgggtttccaaagtgcccccGGGGGCcgtgatatacctgatTGGAGTctctatagctctaaatagGGGTGCacagtacaaatcttcaaaaaggtgtga
D11	S471, T479, T486	AWW	REV	aatttgggtttccaaagtgcccccCGGCGTcgtgatatacctgatTGGAGTctctatagctctaaatagGGGTGCacagtacaaatcttcaaaaaggtgtga
D12	S471, T479, T486	WAA	REV	aatttgggtttccaaagtgcccccGGGGGCcgtgatatacctgatAGGAGCctctatagctctaaatagCGGAGAacagtacaaatcttcaaaaaggtgtga
D13	S471, T479, T486	WAW	REV	aatttgggtttccaaagtgcccccCGGCGTcgtgatatacctgatAGGAGCctctatagctctaaatagCGGAGAacagtacaaatcttcaaaaaggtgtga
D14	S471, T479, T486	WWA	REV	aatttgggtttccaaagtgcccccGGGGGCcgtgatatacctgatTGGAGTctctatagctctaaatagCGGAGAacagtacaaatcttcaaaaaggtgtga
D15	S471, T479, T486	WWW	REV	aatttgggtttccaaagtgcccccCGGCGTcgtgatatacctgatTGGAGTctctatagctctaaatagCGGAGAacagtacaaatcttcaaaaaggtgtga
D16	S471, T479, T486	WWE	REV	aatttgggtttccaaagtgcccccTTCCTCcgtgatatacctgatTGGAGTctctatagctctaaatagCGGAGAacagtacaaatcttcaaaaaggtgtga
D17	S471, T479, T486	WEW	REV	aatttgggtttccaaagtgcccccCGGCGTcgtgatatacctgatCTCTTCctctatagctctaaatagCGGAGAacagtacaaatcttcaaaaaggtgtga
D18	S471, T479, T486	WEE	REV	aatttgggtttccaaagtgcccccTTCCTCcgtgatatacctgatCTCTTCctctatagctctaaatagCGGAGAacagtacaaatcttcaaaaaggtgtga
D19	S471, T479, T486	EWW	REV	aatttgggtttccaaagtgcccccCGGCGTcgtgatatacctgatTGGAGTctctatagctctaaatagTTCCTCacagtacaaatcttcaaaaaggtgtga
D20	S471, T479, T486	EWE	REV	aatttgggtttccaaagtgcccccTTCCTCcgtgatatacctgatTGGAGTctctatagctctaaatagTTCCTCacagtacaaatcttcaaaaaggtgtga
D21	S471, T479, T486	EEW	REV	aatttgggtttccaaagtgcccccCGGCGTcgtgatatacctgatCTCTTCctctatagctctaaatagTTCCTCacagtacaaatcttcaaaaaggtgtga
E1	S496	A	FWD	ggcactttggaaacccaaattGCTCCTagaaagtcctctgcacccgat
E2	S496	E	FWD	ggcactttggaaacccaaattGAGGAGagaaagtcctctgcacccgat
E3	S496	W	FWD	ggcactttggaaacccaaattTCACCAagaaagtcctctgcacccgat
F1	N/A	N/A	REV	tgtgaggacatcgggtgcagaggactttct
HCM1-PMF	N/A	N/A	FWD	CCTTCCTCTCATGGTTCGGA
HCM1-PMR	N/A	N/A	REV	TGTGAGGACATCGGGTGC

Supplementary Table 4. Oligonucleotides for sequencing library construction.

Name	Truseq index number	Truseq index sequence	Orientation	Sequence
MC71	Universal	N/A	FWD	AATGATACGGCGACCACCGAGATCTACACTCTTTCCCTACACGACGCTCTTCCGATCTCTCATGGTTCGGACTTACTT
MC72	1	ATCACG	REV	CAAGCAGAAGACGGCATACGAGAT**CGTGAT**GTGACTGGAGTTCAGACGTGTGCTCTTCCGATCTGGGTGCAGAGGACTTTCT
MC73	2	CGATGT	REV	CAAGCAGAAGACGGCATACGAGAT**ACATCG**GTGACTGGAGTTCAGACGTGTGCTCTTCCGATCTGGGTGCAGAGGACTTTCT
MC80	3	TTAGGC	REV	CAAGCAGAAGACGGCATACGAGAT**GCCTAA**GTGACTGGAGTTCAGACGTGTGCTCTTCCGATCTGGGTGCAGAGGACTTTCT
MC81	4	TGACCA	REV	CAAGCAGAAGACGGCATACGAGAT**TGGTCA**GTGACTGGAGTTCAGACGTGTGCTCTTCCGATCTGGGTGCAGAGGACTTTCT
MC82	5	ACAGTG	REV	CAAGCAGAAGACGGCATACGAGAT**CACTGT**GTGACTGGAGTTCAGACGTGTGCTCTTCCGATCTGGGTGCAGAGGACTTTCT
MC83	6	GCCAAT	REV	CAAGCAGAAGACGGCATACGAGAT**ATTGGC**GTGACTGGAGTTCAGACGTGTGCTCTTCCGATCTGGGTGCAGAGGACTTTCT
MC84	7	CAGATC	REV	CAAGCAGAAGACGGCATACGAGAT**GATCTG**GTGACTGGAGTTCAGACGTGTGCTCTTCCGATCTGGGTGCAGAGGACTTTCT
MC85	8	ACTTGA	REV	CAAGCAGAAGACGGCATACGAGAT**TCAAGT**GTGACTGGAGTTCAGACGTGTGCTCTTCCGATCTGGGTGCAGAGGACTTTCT
MC86	9	GATCAG	REV	CAAGCAGAAGACGGCATACGAGAT**CTGATC**GTGACTGGAGTTCAGACGTGTGCTCTTCCGATCTGGGTGCAGAGGACTTTCT
MC87	10	TAGCTT	REV	CAAGCAGAAGACGGCATACGAGAT**AAGCTA**GTGACTGGAGTTCAGACGTGTGCTCTTCCGATCTGGGTGCAGAGGACTTTCT
MC88	11	GGCTAC	REV	CAAGCAGAAGACGGCATACGAGAT**GTAGCC**GTGACTGGAGTTCAGACGTGTGCTCTTCCGATCTGGGTGCAGAGGACTTTCT
MC89	12	CTTGTA	REV	CAAGCAGAAGACGGCATACGAGAT**TACAAG**GTGACTGGAGTTCAGACGTGTGCTCTTCCGATCTGGGTGCAGAGGACTTTCT
MC90	14	AGTTCC	REV	CAAGCAGAAGACGGCATACGAGAT**GGAACT**GTGACTGGAGTTCAGACGTGTGCTCTTCCGATCTGGGTGCAGAGGACTTTCT
MC91	15	ATGTCA	REV	CAAGCAGAAGACGGCATACGAGAT**TGACAT**GTGACTGGAGTTCAGACGTGTGCTCTTCCGATCTGGGTGCAGAGGACTTTCT
MC92	16	CCGTCC	REV	CAAGCAGAAGACGGCATACGAGAT**GGACGG**GTGACTGGAGTTCAGACGTGTGCTCTTCCGATCTGGGTGCAGAGGACTTTCT

MC93	19	GTGAAA	REV	CAAGCAGAAGACGGCATACGAGAT**TTTCAC**GTGACTGGAGTTCAGACGTGTGCTCTTCCGATCTGGGTGCAGAGGACTTTCT
MC94	20	GTGGCC	REV	CAAGCAGAAGACGGCATACGAGAT**GGCCAC**GTGACTGGAGTTCAGACGTGTGCTCTTCCGATCTGGGTGCAGAGGACTTTCT
MC95	21	GTTTCG	REV	CAAGCAGAAGACGGCATACGAGAT**CGAAAC**GTGACTGGAGTTCAGACGTGTGCTCTTCCGATCTGGGTGCAGAGGACTTTCT
MC96	22	CGTACG	REV	CAAGCAGAAGACGGCATACGAGAT**CGTACG**GTGACTGGAGTTCAGACGTGTGCTCTTCCGATCTGGGTGCAGAGGACTTTCT
MC97	23	GAGTGG	REV	CAAGCAGAAGACGGCATACGAGAT**CCACTC**GTGACTGGAGTTCAGACGTGTGCTCTTCCGATCTGGGTGCAGAGGACTTTCT
MC98	25	ACTGAT	REV	CAAGCAGAAGACGGCATACGAGAT**ATCAGT**GTGACTGGAGTTCAGACGTGTGCTCTTCCGATCTGGGTGCAGAGGACTTTCT
HCM1-PMF	N/A	N/A	FWD	CCTTCCTCTCATGGTTCGGA
MC70	N/A	N/A	REV	CAAGGGATTGGGGATTCCAT
