# Supplementary material for: Phosphosite Scanning reveals a complex phosphorylation code underlying CDK-dependent activation of Hcm1
Source: Nat Commun. 2023 Jan 19;14:310. doi: 10.1038/s41467-023-36035-9 (PMC9852432; doi:10.1038/s41467-023-36035-9)
Supplement: Supplementary file 3 — Description of Additional Supplementary Files [file 41467_2023_36035_MOESM3_ESM.pdf]

Description of Additional Supplementary Files

Supplementary Data 1: Count tables for all screens.

Supplementary Data 2: Primary data for heatmaps.

Supplementary Data 3: Selection coefficients for all screens.
