# Supplementary material for: Phosphosite Scanning reveals a complex phosphorylation code underlying CDK-dependent activation of Hcm1
Source: Nat Commun. 2023 Jan 19;14:310. doi: 10.1038/s41467-023-36035-9 (PMC9852432; doi:10.1038/s41467-023-36035-9)
Supplement: Supplementary file 7 — Reporting Summary [file 41467_2023_36035_MOESM7_ESM.pdf]

nature portfolio

Corresponding author(s): Jennifer Benanti

Last updated by author(s): Dec 29, 2022

Reporting Summary

Nature Portfolio wishes to improve the reproducibility of the work that we publish. This form provides structure for consistency and transparency in reporting. For further information on Nature Portfolio policies, see our Editorial Policies and the Editorial Policy Checklist.

Statistics

For all statistical analyses, confirm that the following items are present in the figure legend, table legend, main text, or Methods section.

n/a	Confirmed	
☐	☒	The exact sample size (*n*) for each experimental group/condition, given as a discrete number and unit of measurement
☐	☒	A statement on whether measurements were taken from distinct samples or whether the same sample was measured repeatedly
☐	☒	The statistical test(s) used AND whether they are one- or two-sided<br>*Only common tests should be described solely by name; describe more complex techniques in the Methods section.*
☒	☐	A description of all covariates tested
☐	☒	A description of any assumptions or corrections, such as tests of normality and adjustment for multiple comparisons
☐	☒	A full description of the statistical parameters including central tendency (e.g. means) or other basic estimates (e.g. regression coefficient) AND variation (e.g. standard deviation) or associated estimates of uncertainty (e.g. confidence intervals)
☐	☒	For null hypothesis testing, the test statistic (e.g. *F*, *t*, *r*) with confidence intervals, effect sizes, degrees of freedom and *P* value noted<br>*Give P values as exact values whenever suitable.*
☒	☐	For Bayesian analysis, information on the choice of priors and Markov chain Monte Carlo settings
☒	☐	For hierarchical and complex designs, identification of the appropriate level for tests and full reporting of outcomes
☒	☐	Estimates of effect sizes (e.g. Cohen's *d*, Pearson's *r*), indicating how they were calculated

Our web collection on statistics for biologists contains articles on many of the points above.

Software and code

Policy information about availability of computer code

Data collection	GuavaSoft (v3.3) was used for collecting flow cytometry data.
Data analysis	GraphPad Prism (v9.4.1) was used for statistical analyses. FlowJo (v10.8.1) was used to analyze flow cytomentry data. IUPred2 was used to generate disorder plots. RStudio 2022.02.2+485 and python3 were used to generate count tables from sequencing data and to make box plots. Custom scripts used to generate count tables for all screens are publicly available on GitHub at https://github.com/radio1988/mutcount and Zenodo at https://doi.org/10.5281/zenodo.7492458.

For manuscripts utilizing custom algorithms or software that are central to the research but not yet described in published literature, software must be made available to editors and reviewers. We strongly encourage code deposition in a community repository (e.g. GitHub). See the Nature Portfolio guidelines for submitting code & software for further information.

Data

Policy information about availability of data

All manuscripts must include a data availability statement. This statement should provide the following information, where applicable:

- Accession codes, unique identifiers, or web links for publicly available datasets
- A description of any restrictions on data availability
- For clinical datasets or third party data, please ensure that the statement adheres to our policy

All sequencing data in this study have been deposited in the NCBI Sequence Read Archive under BioProject # PRJNA841829 [https://www.ncbi.nlm.nih.gov/sra/?term=PRJNA841829]. Source data are provided with this paper.

Human research participants

Policy information about studies involving human research participants and Sex and Gender in Research.

Reporting on sex and gender	N/A
Population characteristics	N/A
Recruitment	N/A
Ethics oversight	N/A

Note that full information on the approval of the study protocol must also be provided in the manuscript.

Field-specific reporting

Please select the one below that is the best fit for your research. If you are not sure, read the appropriate sections before making your selection.

[x] Life sciences [ ] Behavioural & social sciences [ ] Ecological, evolutionary & environmental sciences

For a reference copy of the document with all sections, see nature.com/documents/nr-reporting-summary-flat.pdf

Life sciences study design

All studies must disclose on these points even when the disclosure is negative.

Sample size	Sample size was not predetermined. All screens and experiments were performed at least three times to ensure results were robust and reproducible, as is common in the field.
Data exclusions	No data were excluded.
Replication	All experiments and screens were repeated at least 3 times. All attempts at replication were successful.
Randomization	Mutants that were directly compared were analyzed in parallel in individual experiments and screens, making further randomization not applicable.
Blinding	Mutants analyzed in fitness screens were blinded since they were carried out in pools and analyzed computationally using identical methods. Other experiments do not require blinding because results were directly linked with the data and they are neither a clinical study with large cohorts nor genetics studies with large numbers of samples. Automated data analysis was used whenever possible to reduce potential bias.

Reporting for specific materials, systems and methods

We require information from authors about some types of materials, experimental systems and methods used in many studies. Here, indicate whether each material, system or method listed is relevant to your study. If you are not sure if a list item applies to your research, read the appropriate section before selecting a response.

Materials & experimental systems

n/a	Involved in the study
☐	☒ Antibodies
☒	☐ Eukaryotic cell lines
☒	☐ Palaeontology and archaeology
☒	☐ Animals and other organisms
☒	☐ Clinical data
☒	☐ Dual use research of concern

Methods

n/a	Involved in the study
☒	☐ ChIP-seq
☐	☒ Flow cytometry
☒	☐ MRI-based neuroimaging

Antibodies

Antibodies used

V5 - Invitrogen, Catalog# R960-25, Lot# 1923773, 2024280, diluted 1:1000
HA- 12CA5, gift from David Toczyski, purified from hybridoma, diluted 1:1000
PSTAIRE- Sigma, Catalog# P7962, Lot# 015M4840V, diluted 1:10000

Validation

All antibodies were used in Western blotting. The specificity of antibodies against V5 and HA epitope tags were previously validated in the lab by comparing lysates from yeast strains with each tag to a yeast strain lacking the tags as a negative control. The manufacturer's website states that the PSTAIRE antibody was raised against a 16 amino acid sequence conserved in all CDKs in every eukaryote examined. It has been previously used extensively for Western blotting, ELISA assays and immunopreciptiation. In this study it recognized two bands of the expected sizes and was exclusively used as a loading control so no further validation was performed.

Flow Cytometry

Plots

Confirm that:

☒ The axis labels state the marker and fluorochrome used (e.g. CD4-FITC).

☒ The axis scales are clearly visible. Include numbers along axes only for bottom left plot of group (a 'group' is an analysis of identical markers).

☒ All plots are contour plots with outliers or pseudocolor plots.

☒ A numerical value for number of cells or percentage (with statistics) is provided.

Methodology

Sample preparation

All samples are yeast cells cultured in the lab. For pairwise competition assays, cells were stored in sodium citrate buffer (50mM sodium citrate, 0.02% NaN3, pH 7.4) and then sonicated prior to measuring GFP levels on the cytometer. For cell cycle analysis, cells were fixed in 70% ethanol. Fixed cells were sonicated, treated with 0.25mg/mL RNase A at 50 degrees C for one hour, then subsequently treated with 0.125mg/mL Proteinase K at 50 degrees C for one hour. Cells were then labeled with 1µM Sytox Green (Invitrogen) and analyzed on the cytometer.

Instrument

Millipore Guava easyCyte 5HT

Software

Data was collected using GuavaSoft (v3.3) software. Data was analyzed with FlowJo software (v10.8.1).

Cell population abundance

No sorting was performed.

Gating strategy

For pairwise competition experiments, no gating was preformed. GFP positive and negative populations were clearly separable and the two populations were quantified in each culture at each time point. An representative plot is shown in Source Data for Fig 1c. For the cell cycle time course in Supplementary Fig 1b, FSC/SSC was used to gate out debris. A representative plot showing the gating is shown in the Source Data for Sup Fig 1b. After gating, hstograms showing Sytox Green staining at each time point from the experiment in Supplementary Fig 1a were plotted. These plots were not quantified.

☒ Tick this box to confirm that a figure exemplifying the gating strategy is provided in the Supplementary Information.
